# Natural Compound Methyl Protodioscin Suppresses Proliferation and Inhibits Glycolysis in Pancreatic Cancer

**DOI:** 10.1155/2018/7343090

**Published:** 2018-03-15

**Authors:** LianYu Chen, Chien-shan Cheng, HuiFeng Gao, Ling Zhan, FengJiao Wang, Chao Qu, Ye Li, Peng Wang, Hao Chen, ZhiQiang Meng, LuMing Liu, HaiFeng Chen, Zhen Chen

**Affiliations:** ^1^Department of Oncology, Shanghai Medical College, Fudan University, Shanghai 200032, China; ^2^Department of Integrative Oncology, Fudan University Shanghai Cancer Center, Shanghai 200032, China; ^3^School of Pharmaceutical Sciences, Xiamen University, Xiamen 361000, China

## Abstract

Methyl protodioscin (MPD) is one of the main bioactive components in the plant of Dioscoreaceae. MPD has been demonstrated to possess antitumor activities. However, its role in pancreatic cancer and the underlying molecular mechanisms are poorly defined. In the present study, we demonstrated that MPD inhibited proliferation and promoted apoptosis of pancreatic cancer. Furthermore, our results demonstrated that MPD decreased oncogene c-Myc in protein level and resulted in concomitant reduction in glycolysis. In vivo assays with xenograft mouse model further confirmed the in vitro observations, which indicated that MPD inhibited 18FDG uptake in tumors formed by subcutaneously injection of MIA PaCa-2 cells. Collectively, our present study uncovered novel antitumor functions of MPD in pancreatic cancer and provided the possible molecular mechanism.

## 1. Introduction

Pancreatic cancer is a highly lethal malignant disease because of its aggressive growth and high metastatic rate at early stage [1]. Nowadays, pancreatic cancer is the 4th (for females) or the 5th (for males) leading cause of death from cancer in the western world and its five-year survival rate is less than 6% [2]. Surgical resection is the only curative treatment for pancreatic cancer patients, but only about 15% is suitable at the time of diagnose; chemotherapy and radiation are wildly used to treat inoperable pancreatic cancer patients, but most patients resistant to them [3, 4]. Therefore, additional therapeutic agents should be evaluated to improve the treatment of pancreatic cancer patients.

Methyl protodioscin (MPD), a bioactive natural compound extracted from the rhizome of* Dioscorea collettii* var.* hypoglauca* (Dioscoreaceae), has been investigated for its numerous pharmacological activities, including those related to lipid-lowering, anti-inflammation, and anticancer activities [5–7]. Its anticancer activities have been tested by the National Cancer Institute's (NCI) anticancer drug discovery screen with a panel of 60 human cancer cell lines [8]. It was reported that MPD had distinct cytotoxic activity and lead to G2/M arrest and apoptosis of K562, HepG2, and A549 in vitro [9–11]. Furthermore, a preclinical pharmacodynamic study showed that high dosage (80 mg/kg i.v.) had no serious side effects and MPD has a clear pharmacokinetic/pharmacodynamic profile and a defined chemical structure in Traditional Chinese herbal (TCH) [12]. However, little is known about its effect on pancreatic cancer, and the underlying correlative mechanisms remains elusive.

Recent years have witnessed the profound impact of aberrant cancer cell metabolism to malignancies of cancer cells, and cancer cell reprogramming has been considered to be one hallmarks of cancer [13]. In pancreatic cancer, due to the severe hypoxic conditions caused by dense desmoplasia and limited oxygen and nutrient supply, pancreatic cancer cells rely on glycolysis and glutamine metabolism for the requirement of energy and building blocks to meet demands of uncontrolled proliferation [14–17]. Furthermore, genetic mutations that prevalent in pancreatic cancer have also been demonstrated to shift pancreatic cancer cells metabolism [18, 19]. For example, the mutation in Kras has been demonstrated to cause enhanced aerobic glycolysis and glutamine metabolism [20–22]. The underlying molecular mechanism has been attributed to upregulation of master regulators of metabolism, like c-Myc and HIF1*α* (hypoxia inducible factor 1*α*) [23, 24]. Based on the decisive roles of cancer cell metabolism to the formation and maintenance of malignant properties of pancreatic cancer, it came up with the proposal that targeting cancer cell metabolism might be a promising therapeutic target for the treatment of pancreatic cancer. However, the impact of bioactive natural compounds on pancreatic cancer cell metabolism has seldom been discussed before.

In our present study, we demonstrated that MPD possessed antitumor activities against pancreatic cancer and MPD inhibited pancreatic cancer proliferation by interventions of glycolysis. Mechanism studies suggested that MPD decreased aerobic glycolysis by inhibiting the Akt1/c-Myc axis. Collectively, this study uncovered novel functions of MPD and shed light on future investigation for the application of this natural compound in the treatment of pancreatic cancer.

## 2. Materials and Methods

### 2.1. Cell Culture

The human pancreatic cancer cell lines PANC-1 and MIA PaCa-2 cells were obtained from ATCC. PANC-1 cells were grown in DMEM supplemented with 10% FBS (fetal beef serum) (Hyclone, Logan, UT, USA), penicillin (10,000 U/L), and streptomycin (100 mg/L). MIA PaCa-2 cells were cultured in 10% FBS and 2.5% horse serum that supplemented with penicillin and streptomycin. All cells were culture at 37°C in a 5% CO_2_ humidified atmosphere.

### 2.2. Drugs and Reagents

MPD was purchased from Shanghai Shifeng Biological Technology. Working concentrations were then prepared by diluting stock solutions in culture medium immediately before use. MTT were purchased from Sigma (St. Louis, MO, USA). The TRIzol reagent was purchased from Invitrogen Life Technologies (Paisley, Scotland, UK).

### 2.3. MTT Assay

The effects of MPD on cell proliferation of MIA PaCa-2 and PANC-1 cells were assessed using the MTT assay. All cells were harvested and seeded in 24-well plates at 5.0 × 10^4^ cells/well in a final volume of 500 *μ*L. After 24 hours of incubation, drugs were added to duplicate plates at appropriate concentrations. After 48 hours, MTT dye (5 mg/ml) was added to each well. DMSO (150 *μ*L) was added to each well and vortexed at low speed for 10 minutes to fully dissolve the blue crystals. The concentrations required to inhibit cell growth by 50% (IC50) were calculated from the cytotoxicity curves (Graphpad Prism 5), at least 3 independent experiments.

### 2.4. Cell Cycle, Cell Apoptosis

Flow cytometric analysis was conducted to examine cell cycle and apoptosis with the application of propidium iodine (Invitrogen) and human Annexin V-FITC Kit (Invitrogen), respectively, according to the manufacturer's protocol. All observations were reproduced at least three times in independent experiments.

### 2.5. RNA Isolation and Quantitative Real-Time PCR

Total RNA was isolated by using Invitrogen's TRIzol reagent. To obtain cDNA samples, TaKaRa's RrimeScript RT reagent was used for reverse transcription. The expression status of glycolytic genes and *β*-actin were determined by quantitative real-time PCR using an ABI 7900HT Real-Time PCR system (Applied Biosystems, USA). All reactions were run in triplicate. Primer sequences are listed in Table 1.

### 2.6. Protein Extraction and Western Blot Analysis

Cells were washed twice with ice-dole PBS and lysed in RIPA buffer (150 mM NaCl, 1% NP-40, 50 mM Tris/HCl, pH 8.0 and 10% glycerol) supplemented with protease and phosphatase inhibitor cocktail for 10 min. Cell debris was removed by centrifugation at 12000 rpm for 10 min at 4°C. Thermo Pierce® BCA Protein Assay Kit was used to determine the concentration of the total cell lysates. 20 *μ*g total protein lysate was subjected to electrophoresis in denaturing 10% SDS-polyacrylamide gel separation and then transferred to a membrane for subsequent blotting with antibodies. c-Myc antibody was obtained from Abcam (ab32072). Antibodies to BAX and BCL-2 were obtained from ABclonal Biotech (Wuhan, China). Mouse monoclonal antibody against *β*-actin was purchased from Proteintech (60008-1-Ig). PARP1 and Mcl1 antibodies were obtained from Proteintech (13371-1-AP and 16225-1-AP, resp.).

### 2.7. Oxygen Consumption Rate (OCR) and Extracellular Acidification Rate (ECAR)

To assess the impact of MPD on glycolysis of pancreatic cancer cells, cellular mitochondrial function and glycolytic capacity were measured by using the Agilent's Seahorse Bioscience XF96 Extracellular Flux Analyzer, all according to the manufacturer's instructions of Seahorse XF Cell Mito stress test kit or Glycolysis Stress Test Kit. In brief, cells were plated in XF96 Cell Culture Microplates (Seahorse Bioscience) at an initial cellular density of 4 × 10^4^ cells/well the day before determination. Seahorse buffer consists of DMEM medium, phenol red, 25 mM glucose, 2 mM sodium pyruvate, and 2 mM glutamine. For ECAR measurement, 10 mM glucose, 1 *μ*M oligomycin, and 100 mM 2-deoxy-glucose (2-DG) were automatically added to measure ECAR value. After monitoring baseline respiration, 1 *μ*M oligomycin, 1 *μ*M FCCP, and 1 *μ*M rotenone were automatically injected into XF96 Cell Culture Microplates to measure the OCR. The ECAR and OCR values were calculated after normalization to cell number [25].

### 2.8. Animal Models

To perform in vivo animal model study of the impact of MPD on tumor generation capacity, BALB/c-nu mice (4–6 weeks of age, 18–20 g, Shanghai SLAC Laboratory Animal Co., Ltd.) were used. The mice were housed in sterile filter-capped cages. An amount of 1 × 10^6^ MIA PaCa-2 cells in 100 *μ*l phosphate-buffered saline (PBS) were subcutaneously injected. Four weeks after subcutaneous injection, the mice were prepared for micro-PET/CT scanning. All animal experiments were performed according to the guidelines for the care and use of laboratory animals and were approved by IACUC of Fudan University.

### 2.9. Micro-PET/CT Imaging

Micro-PET/CT scans and image analysis were performed using an Inveon micro-PET/CT (Siemens Medical Solution). Each tumor-bearing mouse was injected with 11.1 MBq (300 *μ*Ci) of 18F-FDG via tail vein. Scanning was started 1 h after injection and animals were anesthetized under isoflurane during scanning period. The images were reconstructed using three-dimensional ordered-subset expectation maximization/maximum algorithm. Inveon Research Workplace was used to obtain percentage injected dose per gram (% ID/g) and standardized uptake values (SUV). The maximum SUV (SUVmax) was calculated.

### 2.10. Statistical Analyses

Statistical analyses were performed by SPSS software (version 17.0, IBM Corp., Armonk, NY, USA) using independent *t*-tests (for continuous variables) and Pearson's *χ*^2^ tests (for categorical variables). Statistical significance was based on two-sided *p* values of <0.05.

## 3. Results

### 3.1. The Quantification of MPD Doses on Pancreatic Cancer

**Figure 1:**
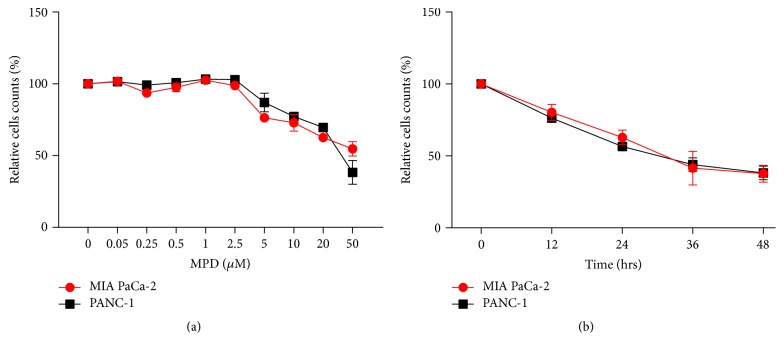
*The quantification of MPD doses on pancreatic cancer* (a and b) MTT assay was performed to quantify the effective doses of MPD on pancreatic cancer cells.

In order to assess the influence of MPD on cell proliferation of pancreatic cancer, MIA PaCa-2 and PANC-1 cells were treated with different concentrations of MPD for different times. MTT assay showed that MPD significantly inhibited growth of MIA PaCa-2 and PANC-1 cells in a time- and dose-dependent manner. The results were presented as a percentage relative to the control cell number. The IC50 values of MIA PaCa-2 and PANC-1 were about 50 and 34.4 *μ*M, respectively (Figures 1(a) and 1(b)).

### 3.2. MPD Inhibited Proliferation of Pancreatic Cancer Cells

**Figure 2:**
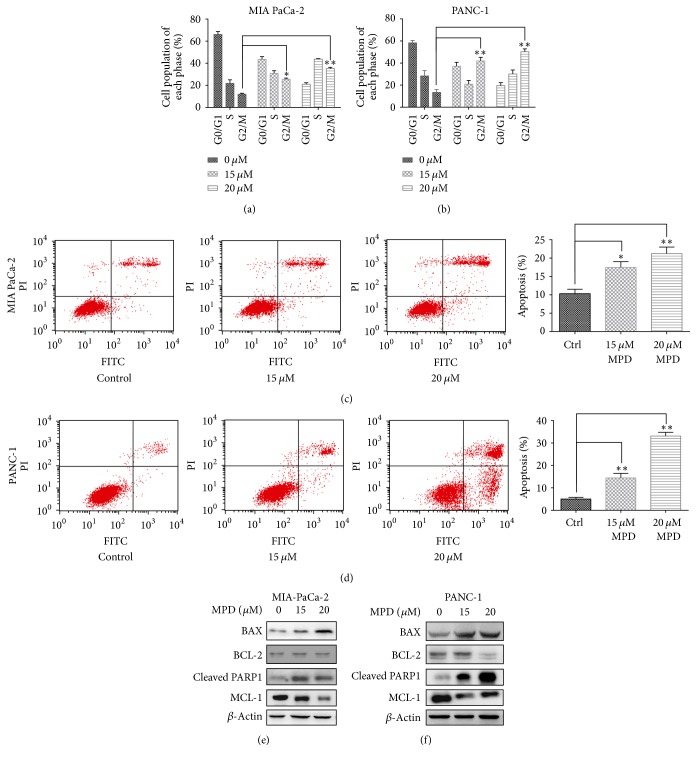
*MPD inhibited proliferation of pancreatic cancer cells.* (a and b) MPD induced cell cycle arrest at G2/M phase in MIA PaCa-2 and PANC-1 cells. (c and d) MPD treatment promoted cell apoptosis in MIA PaCa-2 and PANC-1 cells. (e and f) The effect of MPD on the expression of apoptosis related proteins. ^*∗*^*P* < 0.05, ^*∗∗*^*P* < 0.01.

To examine the impact of MPD on pancreatic cancer cell cycle progression and apoptosis, we carried out flow cytometry analysis. MIA PaCa-2 and PANC-1 cells were treated with various MPD doses and analyzed the DNA content to examine its influence on cell cycle progression. Results indicated that MPD induced a significant cell cycle arrest at G2/M phase following 24-hour treatment with different MPD concentrations (Figures 2(a) and 2(b)). Furthermore, we assessed the impact of MPD on cell apoptosis, and flow cytometry indicated that MPD treatment at a concentration of 15 *μ*M and 20 *μ*M could induce cell apoptosis (Figures 2(c) and 2(d)). In the end, we measured the levels of apoptosis related proteins, including BAX, BCL-2, cleaved PARP1, and Mcl-1. WB results indicated that MPD treatment increased the levels of BAX and cleaved PARP1 in MIA PaCa-2 and PANC-1 cells and decreased expression of antiapoptotic factor Mcl-1 expression (Figures 2(e) and 2(f)).

### 3.3. MPD Inhibited Glycolysis in Pancreatic Cancer Cells

**Figure 3:**
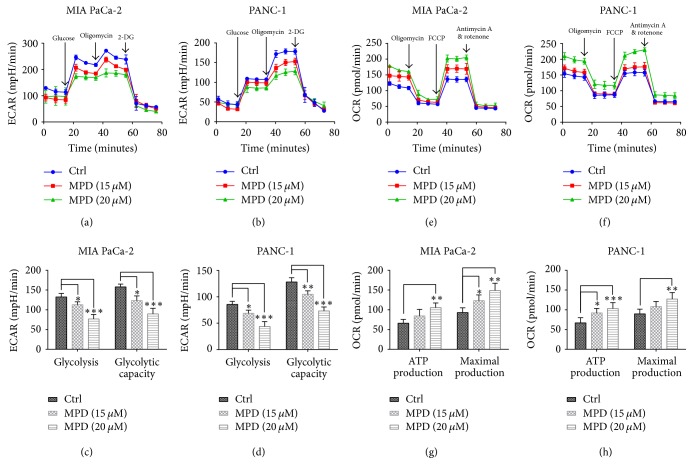
*MPD inhibited glycolysis in pancreatic cancer cells*. (a and b) A representative graph of ECAR outputs from the Seahorse XF analyzer. (c and d) MPD significantly inhibited glycolysis and glycolytic capacity in MIA PaCa-2 and PANC-1 cells. (e and f) A representative of the OCR outputs from the Seahorse XF analyzer. (g and h) MPD significantly increased ATP production and maximal respiration. ^*∗*^*P* < 0.05, ^*∗∗*^*P* < 0.01, ^*∗∗∗*^*P* < 0.001.

Metabolism transformation participated in various malignant behaviors of cancer cells, including uncontrolled proliferation. To ask whether the influence of MPD on decreased cell proliferation was due to its impact on metabolism, we measured the impact of MPD on cell glycolysis, the process that provides cancer cells with ATP, and building blocks for macromolecule synthesis. As is shown, MPD treatment at concentrations of 15 *μ*M and 20 *μ*M decreased glycolysis rate as reflected by ECAR measured by Seahorse energy flux analysis (Figures 3(a) and 3(b)). The impacts of MPD on glycolysis and glycolytic capacity were further analyzed by monitoring changes in ECAR in response to sequential addition of D-glucose to assess glycolysis and oligomycin to measure maximal glycolytic capacity. And our results indicated that MPD could inhibit glycolysis and glycolytic capacity (Figures 3(c) and 3(d)). As observed above, MPD could induce mitochondrial localized apoptotic factor BAX. It is accepted that mitochondria could regulate glucose metabolism. Then we measured the influence of MPD on mitochondrial respiration, which could be assayed by Seahorse analyzer and reflected by OCR. Further mitochondrial stress test, where cells were treated with oligomycin, carbonyl cyanide-p-trifluoromethoxyphenylhydrazone, and rotenone/antimycin to inhibit Complex V, uncouple the proton gradient, and inhibit Complexes I and III, respectively. This allowed for the calculation of basal respiration, ATP production, maximal respiration, and spare respiratory capacity, all measured by changes in oxygen consumption rate. As is shown, MPD treatment could increase the OCR value (Figures 3(e) and 3(f)). Furthermore, MPD inhibited ATP production and maximal respiration significantly (Figures 3(g) and 3(h)). Collectively, these results indicated that MPD could inhibit glycolysis in pancreatic cancer cells.

### 3.4. MPD Negatively Regulated Glycolytic Gene Expression

**Figure 4:**
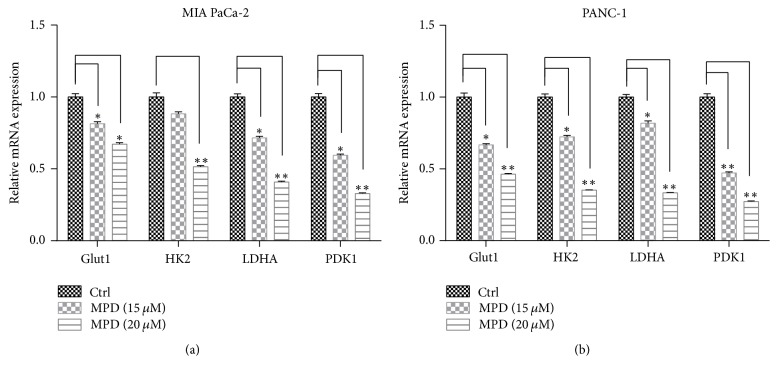
*MPD negatively regulated glycolytic gene expression*. (a and b) MPD treatment decreased the expression status of glycolytic genes, including GLUT1, HK2, LDHA, and PDK1. ^*∗*^*P* < 0.05, ^*∗∗*^*P* < 0.01.

To further validate the impact of MPD on glycolysis, we examined the changes of glycolytic genes upon MPD treatment. As is shown, MPD treatment at concentrations of 15 *μ*M and 20 *μ*M significantly decreased the levels of glycolytic genes including glucose transporter 1 (Glut1), hexokinase 2 (HK2), lactate dehydrogenase A (LDHA), and pyruvate dehydrogenase kinase 1 (PDK1) in MIA PaCa-2 and PANC-1 cells (Figures 4(a) and 4(b)).

### 3.5. MPD Inhibited Akt1/c-Myc Axis in Pancreatic Cancer Cells

**Figure 5:**
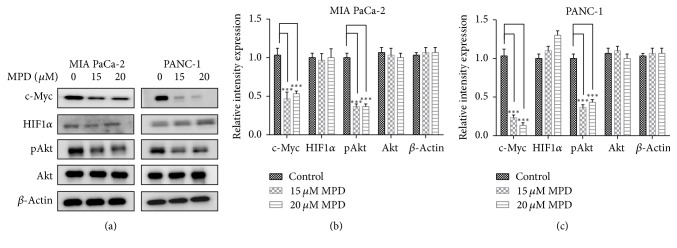
*MPD inhibited Akt1/c-Myc axis in pancreatic cancer cells* (a) MPD treatment deceased the activation status of Akt1 and c-Myc but had slight impact on HIF1*α* expression. (b and c) The band intensities were measured by densitometry and the relative indicated protein expression was shown. ^*∗∗∗*^*P* < 0.001.

To seek for the underlying molecular mechanism that accounted for MPD in regulating glycolysis, we examined the activation status of Akt1. And immunoblotting analysis indicated that MPD inhibited the activation of Akt1. Furthermore, we analyzed changes in c-Myc and HIF1*α* upon MPD treatment, two transcription factors that are responsible for glycolysis and metabolism transformation in cancerous cells. Our results demonstrated that c-Myc decreased significantly, while no changes in HIF1*α* were observed (Figures 5(a)–5(c)). Collectively, these results suggested that MPD might exert its impact on aerobic glycolysis via the Akt1/c-Myc axis.

### 3.6. MPD Inhibited Tumor Formation and Aerobic Glycolysis In Vivo

**Figure 6:**
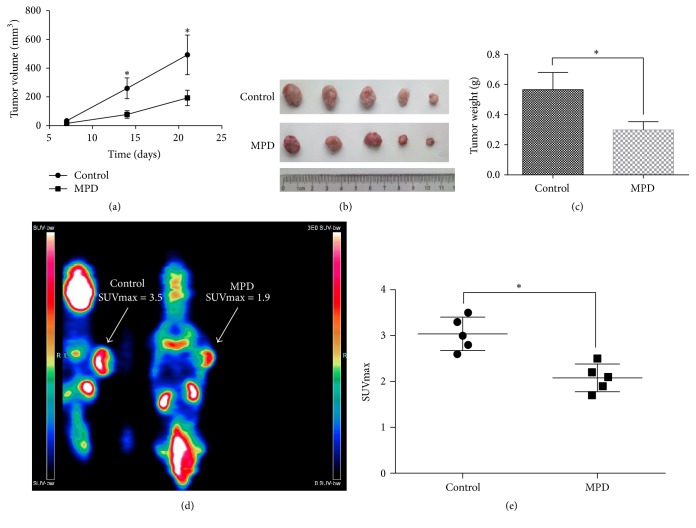
*MPD inhibited tumor formation and aerobic glycolysis in vivo*. (a–c) MPD treatment in subcutaneous mouse model results demonstrated that MPD could inhibit tumor formation capacity of MIA PaCa-2 cells. (d and e) MPD treatment decreased 18FDG uptake in mouse, reinforcing its roles on glycolysis regulation. ^*∗*^*P* < 0.05.

To further confirm the impact of MPD on tumor growth, we performed xenograft mouse model. As illustrated, MPD treatment (100 mg/kg, i.v. injection) significantly inhibited tumor growth and formation capacity of MIA PaCa-2 cells in vivo (Figures 6(a)–6(c)). To validate the observations obtained in vitro, we performed micro-PET/CT Imaging of MIA PaCa-2 subcutaneously injected mouse tumors. As is shown, MPD significantly decreased ^18^FDG uptake in tumors and further supported the observation that MPD could decrease aerobic glycolysis in vivo (Figures 6(d) and 6(e)).

## 4. Discussion

In the present study, we demonstrated that MPD could exert antiproliferative impacts on pancreatic cancer cell lines though inhibiting cell proliferation, promoting apoptosis. Furthermore, to prove the effects of MPD on pancreatic cancer, we performed in vivo injection of MPD and observed that MPD could inhibit tumorigenesis of pancreatic cancer cell lines. To answer the underlying molecular mechanism, our results indicated that MPD could inhibit aerobic glycolysis, a process that provides cancerous cells with the energy and nutrient supply, and MPD intervened the process of glycolysis by inhibiting the Akt1/c-Myc axis.

Due to significant progress in the diagnosis and treatment for pancreatic cancer, the 5-year overall survival remains satisfying and stays steadily at about 6%. Resistance to traditional radiotherapy and chemotherapy makes the improvement in the overall survival disappointing. Thus, it is vital to find and develop novel therapeutic approaches that aids in the prevention and treatment of pancreatic cancer. MPD has been previously demonstrated by NCI (National Cancer Institute, USA) to possess antitumor effects in many tumor cell lines. However, its impact on pancreatic cancer and the underlying molecular mechanism remains elusive. In our present study, we discussed the antitumor effect of MPD on pancreatic cancer cell lines in vitro and in vivo and indicated that MPD could inhibit proliferation and promote apoptosis. These results are in agreement with previous study, demonstrating that MPD could regulate the expression of apoptosis related factor, including BAX and BCL-2. In our present study, we also demonstrated that MPD could decrease the antiapoptosis factor Mcl-1 expression. Mcl-1 is an antiapoptosis factor and has been demonstrated to be re-regulated in many types of cancer and has been reported to be indicators of prognosis and overall survival [26]. Furthermore, Mcl-1 has been reported to participate in chemotherapy and radiotherapy resistance [27]. In patients with Kras mutation backgrounds, overexpression of Mcl-1 in protein levels caused by genetic mutations in Kras and the resultant constitutively activation of ERK signaling has been demonstrated to be a decisive factor for drug resistance [28–30]. Thus, it inspired us to propose that combination of MPD with chemotherapy and radiotherapy might help to improve the inefficacy of traditional treatment strategies [31].

Cancer cell metabolism reprogramming has been regarded as one hallmarks of cancer, and its impact on malignancies of cancer cells has been widely accepted. In pancreatic cancer, severe hypoxic conditions pose a threat to the survival of cancerous cells [32, 33]. To survive under such hostile conditions, pancreatic cancer cell rely on aerobic glycolysis for survival. From the energy-product, the process of glycolysis might be less efficient, as glycolysis produces two ATPs, less than that by mitochondrial phosphorylation. But, through the multisteps of glycolysis, glucose was metabolized to produce building blocks for the synthesis of other macromolecules, including lipid, nucleotide, and amino acid, to meet the demand of uncontrolled proliferation [34]. Furthermore, lactate acid produced by glycolysis creates an acidic microenvironment. Under acidic conditions, the extracellular matrix became less stable and was destructed, favoring cancer cell metastasis [35, 36]. Another contribution of hypoxic glycolysis to pancreatic cancer malignant properties is that it participated to chemotherapy and radiotherapy resistance, by upregulating of reactive oxygen species and HIF1*α* [37, 38]. Thus, it was proposed that targeting pancreatic cancer cell metabolism might be helpful for discovering novel therapeutic targets and pancreatic cancer cells might be starved by “cutting the fuel supply.” In our present study, we demonstrated that MPD could inhibit the glycolysis and suppressed the expression of key glycolysis genes, including Glut1, HK2, and LDHA. These are novel discoveries that have seldom been discussed before. The application and manifestation of glycolysis in cancer are the technique of PET/CT imaging [39]. In our study, we also uncovered the inhibitory roles of MPD on cancer by inhibiting 18FDG uptake, which could be reflected by micro-PET/CT imaging. These observations uncovered novel functions of MPD on pancreatic cancer cell metabolism and might aid in the development of novel treatment targets and strategies for pancreatic cancer.

Cancer cell metabolism is a process that is under the control of many signaling pathways. Among the many cascades, the governing programs converged on c-Myc and HIF1*α*, two master regulators of metabolism [40]. In pancreatic cancer, the genetic aberrations in cancer driver gene Kras were proved to regulate c-Myc and HIF1*α*. Kras mutation could result in constitutive activation of ERK cascade, which phosphorylated the E3 ubiquitin ligase FBW7 and promoted FBW7 degradation. FBW7 was responsible for protein stability of c-Myc and Mcl-1, leading to their upregulation in protein level [41, 42]. Kras could also increase HIF1*α* protein level through posttranslational modifications. In our study, we demonstrated that MPD had slight impact on HIF1*α* but regulated c-Myc protein levels. MPD could inhibit the activation of Akt1 pathway, a decisive factor in governing c-Myc protein level stability. These studies shed light on novel impacts of MPD on signaling transduction in cancer cells. Furthermore, due to the roles of c-Myc to cancer cell proliferation and progression, c-Myc was a druggable target. And many attempts have been made to develop small molecule that could regulate c-Myc. For example, the BET bromodomain inhibitor JQ-1 has been demonstrated to possess antitumor effects in many types of cancer, and the underlying molecular mechanism might in part due to its impact on c-Myc regulation [43]. Thus, combinational use of JQ-1 with MPD might indicate novel treatment strategies.

## 5. Conclusions

In conclusion, our present study sheds light on novel function of MPD in the treatment of pancreatic cancer and provided the possible underlying molecular mechanism. We believe that MPD may point out novel candidates and strategies for the treatment of pancreatic cancer.

## Figures and Tables

**Table 1:** Primer sequences for targeted genes.

Gene	Direction	Sequence (5′-3′)
Glut1	Forward	CTTTGTGGCCTTCTTTGAAGT
	Reverse	CCACACAGTTGCTCCACAT
HK2	Forward	GATTGTCCGTAACATTCTCATCGA
	Reverse	TGTCTTGAGCCGCTCTGAGAT
LDHA	Forward	TGGAGATTCCAGTGTGCCTGTATGG
	Reverse	CACCTCATAAGCACTCTCAACCACC
PDK1	Forward	CTAGAGGGTTACGGGACAGATGCA
	Reverse	CCAAGTGTGTCTAGGCACTGCGGA
*β*-Actin	Forward	CTACGTCGCCCTGGACTTCGAGC
	Reverse	GATGGAGCCGCCGATCCACACGG
